# Comment on “Reverse-sequence endoscopic nipple-sparing mastectomy with direct-to-implant breast reconstruction and air inflation adjustment technique in patients with large or severely ptotic breast: a single-center prospective cohort study”

**DOI:** 10.1097/JS9.0000000000003120

**Published:** 2025-08-07

**Authors:** Jie Peng, Ye Liu, Lichao Zhu, Yating Zhao

**Affiliations:** Breast Disease Treatment Center, Affiliated Hospital of North China University of Science and Technology, Tangshan, Hebei, China

*Dear Editor,*

We read with great interest the article by Dai *et al* titled *“Reverse-sequence endoscopic nipple-sparing mastectomy with direct-to-implant breast reconstruction and air inflation adjustment technique in patients with large or severely ptotic breast: a single-center prospective cohort study”* published in the *International Journal of Surgery* (DOI: 10.1097/JS9.0000000000002389)^[1]^. This prospective cohort study innovatively assesses the feasibility of reverse-sequence endoscopic nipple-sparing mastectomy (R-E-NSM) combined with direct-to-implant breast reconstruction (DIBR) and air inflation adjustment technique (AIAT) in patients with large or severely ptotic breasts (LSPB), a group often considered challenging or contraindicated for conventional open nipple-sparing mastectomy (NSM). By employing propensity score matching (PSM) to compare outcomes between 88 LSPB and 256 non-LSPB (NLSPB) patients, the authors demonstrate comparable surgical safety, aesthetic results, and oncologic outcomes, thereby expanding potential indications for minimally invasive approaches in this population. In line with the TITAN 2025 guideline on AI transparency ^[2]^, a generative AI–based language model was used solely for language polishing and had no bearing on the scientific content.

The study’s key findings highlight the procedure’s viability: after PSM, no significant differences were observed in overall complications (27.3% vs. 22.7%, *P* = 0.381), implant-related issues (21.6% vs. 24.6%, *P* = 0.566), or oncologic metrics such as local recurrence-free survival (LRFS), distant metastasis-free survival (DMFS), and disease-free survival (DFS) (all *P* > 0.05). Notably, AIAT enhanced aesthetic scores in the LSPB group, with higher BREAST-Q satisfaction ratings for breast appearance (*P* = 0.004 when compared to NLSPB without AIAT), suggesting that this adjunctive maneuver – facilitating implant positioning through controlled air inflation – mitigates gravitational challenges in ptotic tissues. These results align with evolving evidence that endoscopic techniques can reduce operative morbidity in larger breasts, contrasting earlier reports of heightened risks in open NSM^[3]^.

Although the study design is robust, incorporating PSM to address confounders like age and tumor stage, several limitations merit attention. Primarily, its single-center execution in a Chinese institution may introduce geographical bias, limiting external validity; for example, demographic factors such as prevalent breast morphologies or comorbidities in Asian cohorts could skew applicability to more heterogeneous global populations, where variations in body habitus or surgical preferences might alter outcomes. Furthermore, the modest sample size (344 patients post-PSM) and median follow-up of approximately 2 years (range: 3.8–52.4 months) may fail to detect infrequent late events, including implant displacement, capsular contracture, or oncologic recurrences that typically manifest beyond this interval. Retrospective analyses of NSM cohorts have reported late complications – defined as those occurring after 1 month postoperatively – at rates up to 34.2%, with capsular contracture affecting 22.2% and prosthesis dislocation 8.2% of cases, often linked to chronic inflammatory processes involving fibroblast proliferation and collagen deposition around the implant, exacerbated by factors such as adjuvant chemotherapy (odds ratio 1.76). Oncologic recurrences, including locoregional relapses (8.5%), frequently emerge later, with 50% diagnosed 8 years post-diagnosis and others between 2 to 5 years, underscoring the need for prolonged surveillance to capture events driven by residual microscopic disease or metastatic progression^[4]^. Additionally, while skin flap necrosis rates did not differ significantly (though observed at 6.8% in LSPB vs. 3.1% in NLSPB, *P* = 0.230), the vulnerability in LSPB patients warrants deeper scrutiny. Literature extensively documents perfusion deficits in thin, elongated skin envelopes of ptotic breasts, driven by multifactorial mechanisms: intraoperative mechanical disruption of subdermal plexuses impairs microvascular integrity, compounded by ptosis-induced chronic venous stasis that heightens susceptibility to ischemia-reperfusion injury. This process activates endothelial proinflammatory pathways, including oxidative stress via reactive oxygen species accumulation and cytokine-mediated inflammation (e.g., elevated TNF-α and IL-6), potentially culminating in flap compromise if postoperative factors like inadequate pressure management or gravitational shear exacerbate hypoperfusion. Such dynamics, as seen in high-risk NSM cohorts, underscore the need for vigilant intraoperative viability assessment to prevent escalation^[5]^.

To elucidate these risks, we recommend investigating flap perfusion mechanisms through histopathological analysis of microvascular changes or biomechanical modeling of AIAT-induced stresses^[6]^. Subgroup stratification by body mass index (BMI) or mastectomy weight could further refine AIAT’s role across ptosis severities.

We advocate for multicenter randomized trials with extended follow-up to validate long-term quality-of-life impacts and compare R-E-NSM against robotic NSM or open methods, including cost-benefit analyses.

Clinically, this approach offers a promising alternative for LSPB patients, potentially minimizing morbidity while preserving natural aesthetics, provided surgeons account for the technical learning curve and institutional resources.

In conclusion, we commend the authors for their insightful work and look forward to additional studies refining this technique.

## Data Availability

No new data were generated or analyzed.

## AI assistance

A generative AI–based language model was employed solely for language polishing.

## References

[1] Dai H Cao X Wu H. Reverse-sequence endoscopic nipple-sparing mastectomy with direct-to-implant breast reconstruction and air inflation adjustment technique in patients with large or severely ptotic breast: a single-center prospective cohort study. Int J Surg 2025;111:3838–49. 40214254 10.1097/JS9.0000000000002389 PMC12165474

[2] Agha RA Mathew G Rashid R. Transparency in the reporting of artificial intelligence – the TITAN guideline. Premier J Sci 2025;10:100082.

[3] Frey JD Salibian AA Karp NS Choi M. The impact of mastectomy weight on reconstructive trends and outcomes in nipple-sparing mastectomy: progressively greater complications with larger breast size. Plast Reconstr Surg 2018;141:795e–804e. 10.1097/PRS.0000000000004404 29794693

[4] Metere A Fabiani E Lonardo MT Giannotti D Pace D Giacomelli L. Nipple-sparing mastectomy long-term outcomes: early and late complications. Medicina (Kaunas) 2020;56:166. 32276470 10.3390/medicina56040166 PMC7230840

[5] Tondu T Hubens G Tjalma WA. Breast reconstruction after nipple-sparing mastectomy in the large and/or ptotic breast: a systematic review of indications, techniques, and outcomes. J Plast Reconstr Aesthet Surg 2020;73:469–85. 31987776 10.1016/j.bjps.2019.11.047

[6] Martinovic ME Pellicane JV Blanchet NP. Surgical delay of the nipple-areolar complex in high-risk nipple-sparing mastectomy reconstruction. Plast Reconstr Surg Glob Open 2016;4:e760. 27482499 10.1097/GOX.0000000000000716 PMC4956872

